# FSN: Joint Entity and Relation Extraction Based on Filter Separator Network

**DOI:** 10.3390/e26020162

**Published:** 2024-02-12

**Authors:** Qicai Dai, Wenzhong Yang, Fuyuan Wei, Liang He, Yuanyuan Liao

**Affiliations:** 1 School of Computer Science and Technology, Xinjiang University, Urumqi 830017, China; 107552101304@stu.xju.edu.cn (Q.D.); wfy@stu.xju.edu.cn (F.W.); heliang@tsinghua.edu.cn (L.H.); liaoyuan@xju.edu.cn (Y.L.); 2 Xinjiang Key Laboratory of Multilingual Information Technology, Xinjiang University, Urumqi 830017, China; 3 Department of Electronic Engineering, Tsinghua University, Beijing 100084, China

**Keywords:** subtask interaction balance, local feature extraction, dynamic loss function, BERT

## Abstract

Joint entity and relation extraction methods have attracted an increasing amount of attention recently due to their capacity to extract relational triples from intricate texts. However, most of the existing methods ignore the association and difference between the Named Entity Recognition (NER) subtask features and the Relation Extraction (RE) subtask features, which leads to an imbalance in the interaction between these two subtasks. To solve the above problems, we propose a new joint entity and relation extraction method, FSN. It contains a Filter Separator Network (FSN) module that employs a two-direction LSTM to filter and separate the information contained in a sentence and merges similar features through a splicing operation, thus solving the problem of the interaction imbalance between subtasks. In order to better extract the local feature information for each subtask, we designed a Named Entity Recognition Generation (NERG) module and a Relation Extraction Generation (REG) module by adopting the design idea of the decoder in Transformer and average pooling operations to better capture the entity boundary information in the sentence and the entity pair boundary information for each relation in the relational triple, respectively. Additionally, we propose a dynamic loss function that dynamically adjusts the learning weights of each subtask in each epoch according to the proportionality between each subtask, thus narrowing down the difference between the ideal and realistic results. We thoroughly evaluated our model on the SciERC dataset and the ACE2005 dataset. The experimental results demonstrate that our model achieves satisfactory results compared to the baseline model.

## 1. Introduction

Joint entity and relation extraction aims at extracting both entities and relations from a given text and finally connecting the semantic links between entities through relations, presenting the relation triples in the text in the form of (s, r, o). As subtasks of information extraction, joint entity and relation extraction provide theoretical and technical support for many research areas, such as knowledge graph construction [1], text summarization [2], and question answering [3].

The majority of the early research on Named Entity Recognition (NER) and Relation Extraction (RE) was realized through pipeline-based methods, such as the models proposed by Zelenko et al. [4] in 2002, Zhou et al. [5] in 2005, and Chan and Roth et al. [6] in 2011. However, this approach has two fatal drawbacks. First, it separates the two subtasks of NER and RE without taking into account the interaction between these two subtasks. Second, this method generally performs the NER task before the RE task, so it is susceptible to receiving the effect of error propagation [7].

**Figure 1:**
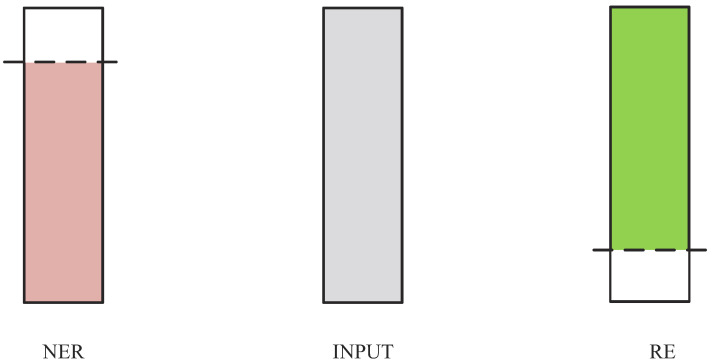
Subtask feature distribution. Pink represents the distribution of Named Entity Recognition (NER) features in the input features. Green represents the distribution of Relation Extraction (RE) features in the input features.

In order to address problems that are difficult to solve with conventional pipeline-based methods, researchers have begun to explore joint entity and relation extraction methods, such as the models proposed by Yan et al. [8] in 2021, Ma et al. [9] in 2022, and Ma et al. [10] in 2022. Although these methods have made much progress in joint entity and relation extraction, they ignore the association and difference between the NER subtask features and the RE subtask features, which leads to an imbalance in the interaction between these two subtasks. As shown in Figure 1, the NER subtask features and RE subtask features have partial overlap in the input features. If these two features are not effectively separated, it can lead to the over-training of one subtask and the inadequate extraction of features for the other subtask.

Therefore, in order to address the above issues, we propose a new joint entity and relation extraction method, FSN. In order to balance the subtask interactions, we set up a Filter Separation Network (FSN) module, which first filters out the hidden state information and the memory state information in the sentence through the LSTM in both directions, and then separates the fused state information of the sentence into the features that are only related to the NER, the features shared by the two subtasks, and the features that are only related to the RE through the separation operation. Finally, the features related to the NER task and the features related to the RE task are obtained through the stitching operation. In order to be able to better extract the local feature information of the two subtasks separately, by adopting the idea of decoder construction in Transformer and pooling operations, we designed a Named Entity Recognition Generation (NERG) module to capture the boundary information of all entities in a sentence as well as a Relation Extraction Generation (REG) module to capture the entity pair boundary information corresponding to each relation in a sentence. We evaluated our model on the ACE2005 and SciERC datasets. Numerous experiments demonstrate that our model outperforms other models.

In summary, our contribution is as follows:

(1) We propose a FSN module that employs a two-directional LSTM to filter and separate the information contained in sentences as well as a splicing operation to merge similar features, thus solving the problem of interaction imbalance between subtasks in joint entity and relation extraction.
(2) We propose a NERG module and a REG module, which better capture the boundary information of all entities in a sentence and the entity pair boundary information corresponding to each relation in a sentence, respectively, by adopting pooling operations and the design ideas of the decoder in Transformer, thus enabling better extraction of local feature information for each subtask.
(3) We propose a dynamic loss function that dynamically adjusts the learning weights of each subtask in each epoch according to the proportionality of losses between each subtask, thus narrowing down the difference between the ideal and realistic results.
(4) We conducted extensive experiments on the ACE2005 and SciERC datasets, which demonstrated that our method achieves better results compared to the baseline model. Further ablation studies and analyses confirm the validity of each module construct in our model.

## 2. Related Work

The majority of the early research used a pipeline-based method before exploring joint entity and relation extraction methods, such as those utilized by Zelenko et al. [4] in 2002, Zhou et al. [5] in 2005, and Chan and Roth et al. [6] in 2011. This method can be separated into two different tasks: NER and RE. It initially extracts every entity from the input text before predicting the relations between every pair of entities. Nevertheless, this method suffers from two significant flaws. First, it divides the two tasks of NER and RE without taking into account their interaction, and second, it is susceptible to mistake propagation [7].

In order to address the issues of conventional pipeline-based methods, researchers have begun to explore joint entity and relation extraction methods. These can be divided into two main categories: feature engineering-based methods and neural network- based methods.

The feature engineering-based method first transforms the raw data into features that express the essence of the problem and then applies these features to the model to improve the model performance, such as in the models proposed by Kate et al. [11] in 2010, Yu et al. [12] in 2010, Miwa et al. [13] in 2014, and so on. However, this method relies heavily on Natural Language Processing (NLP) tools in the process of acquiring features, requires a large amount of manpower and specialized domain knowledge, and suffers from the same problem of error propagation, which ultimately affects the results of joint extraction.

Due to the excellent feature learning ability of neural networks [14], neural network-based methods are gradually applied to joint entity and relation extraction. We categorize these methods into two primary categories based on the research lines adopted by the current neural network-based methods.

**Shared parameters-based methods.** These methods allow each subtask to have an independent decoder, and information interaction is achieved by letting subtasks share sequence-encoding information among themselves, such as the models proposed by Miwa et al. [15] in 2016, Dai et al. [16] in 2019, Yuan et al. [17] in 2020, Shen et al. [18] in 2021, Xiong et al. [19] in 2022, and so on. However, it is exceptionally difficult for such methods to explore the interaction between two subtasks in depth.

**Joint decoding-based method.** This method usually superimposes a unified decoder on the sequence coding layer, which is directly decoded to obtain the relational triple information. Examples include the models proposed by Wang et al. [20] in 2020, Ren et al. [21] in 2021, Yan et al. [8] in 2021, Ma et al. [9] in 2022, Ma et al. [10] in 2022, and so on. However, this method requires the design of complex decoding architectures, which prevents each subtask from adequately extracting local features.

It can be seen that both of the above methods have fatal flaws and cannot effectively solve the problem proposed in this paper. Therefore, in order to solve the above problem, we designed a filter separation network. It first filters the hidden state information as well as the memory state information from each word to the next word in the forward and reverse directions of the sentence, then adopts the idea of partitioning to classify the fusion state information into features related to NER only, features shared by the two subtasks, and features related to RE only, and finally realizes the interaction balance of the two subtasks through the splicing operation. In addition, we designed the NERG and REG modules to further capture the local feature information in the NER and RE tasks, respectively. We conducted extensive experiments on the ACE2005 and SciERC datasets, and the experimental results demonstrate the validity of our model design.

## 3. Methodology

**Figure 2:**
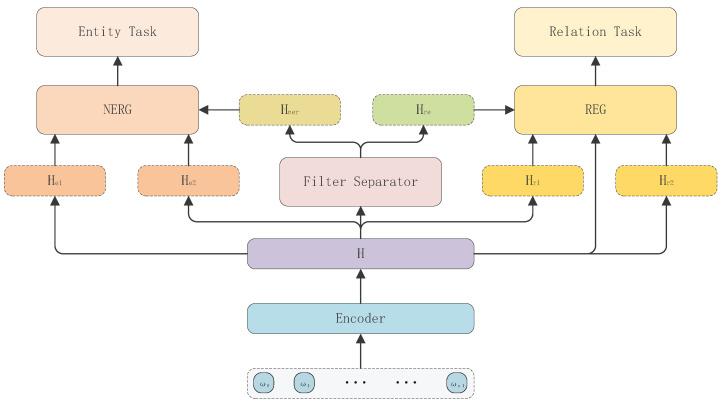
General model architecture.

We will describe our model design in this section. The general structure of our model is shown in Figure 2, which consists of an Encoder module, a Filter Separator Network (FSN) module, a Named Entity Recognition Generation (NERG) module, and a Relation Extraction Generation (REG) module. For each given sentence S = $\omega_{1}\omega_{2}\ldots \omega_{n}$, we first generate the sentence representation through the Encoder module, then feed the sentence representation to the FSN module to obtain the information related to NER and the information related to RE, and then finally feed these two kinds of information into the NERG module and the REG module, respectively, so as to complete the extraction of the entities in the sentence as well as the relation triples.

### 3.1. Encoder Module

Here, we use the pre-trained model BERT-Base-Cased [22] as an encoder for our model. For each given sentence, the module first encodes the sentence into a sequence of token representations (notated as $H\in R^{n\times d_{n}}$). For the NERG module, we transmit the token representation sequence H generated by the encoder to two independent FFNs (Feed-Forward Networks) to generate the feature $H_{e1}$ representing the start boundary of the entity and the feature $H_{e2}$ representing the end boundary of the entity, respectively, as expressed in Equation (1).

(1) $$\begin{array}{cc} \; & H_{e}1=W_{e}1H+b_{e}1\\ \; & H_{e}2=W_{e}2H+b_{e}2 \end{array}$$

where $W_{e1/e2}\in R^{d_{h}\times d_{h}}$ and $b_{e1/e2}\in R^{d_{h}}$ are trainable weights and biases, respectively.

For the REG module, we send the token representation sequence H generated by the encoder to two independent FFNs (Feed-Forward Networks) to generate the feature $H_{r1}$ representing the start boundary of the entity pair and the feature $H_{r2}$ representing the end boundary of the entity pair, respectively, as expressed in Equation (2).

(2) $$\begin{array}{cc} \; & H_{r}1=W_{r}1H+b_{r}1\\ \; & H_{r}2=W_{r}2H+b_{r}2 \end{array}$$

where $W_{r1/r2}\in R^{d_{h}\times d_{h}}$ and $b_{r1/r2}\in R^{d_{h}}$ are trainable weights and biases, respectively.

### 3.2. Filter Separator Network (FSN) Module

**Figure 3:**
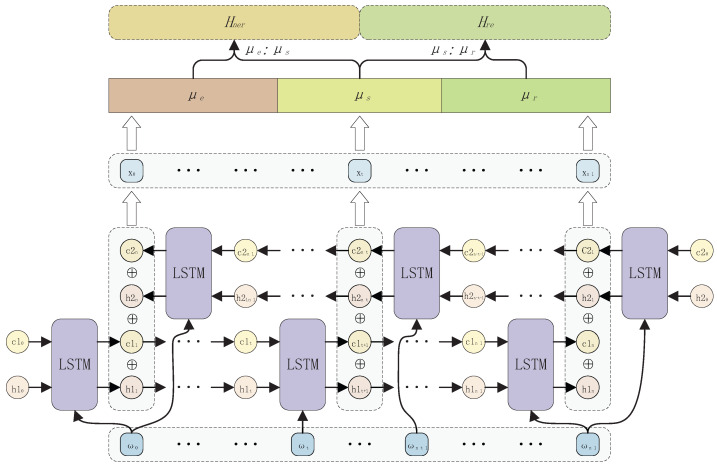
Filter Separator Network (FSN) module.

The structure of the FSN module is shown in Figure 3. The FSN module first utilizes the properties of LSTM to extract the hidden state information and memory state information from each word to the next word in the sentence using LSTM in both directions. Then, the hidden state information and memorized state information obtained by inputting the same word into the LSTM in both directions are fused, thereby obtaining the fused-state feature X = $x_{1}x_{2}\ldots x_{n}$ for the sentence. The separation operation is then used to separate the fusion state into features related only to NER, shared features, and features related only to RE. Finally, we splice the shared features with the features related to NER only and RE only to obtain the features related to NER in the sentence and the features related to RE in the sentence, respectively.

#### 3.2.1. Filter

Since the hidden state information in LSTM captures the information of the current time step and passes this information to the next time step, it enables continuous modeling of sequence data; and the memory state information controls the flow and retention of information, which allows the model to selectively forget and retain the information, thus enabling the capture of long-term dependencies as well as a better prediction of future sequences. Therefore, we use two-direction ground LSTM to capture sentence bi-directional hidden state information as well as memorized state information. The specific formula is shown in Equation (3).

(3) $$\begin{array}{cc} \; & {H1}_{t+1},{C1}_{t+1}=LSTM\left(\omega_{t},{H1}_{t},{C1}_{t}\right)\\ \; & {H2}_{n-t},{C2}_{n-t}=LSTM\left(\omega_{n-t-1},{H2}_{n-t-1},{C2}_{n-t-1}\right) \end{array}$$

where $\omega_{t}$ denotes the i-th word in the sentence S. ${H1}_{t+1}$ and ${C1}_{t+1}$ denote the hidden state information and memorized state information from $\omega_{t}$ to $\omega_{t+1}$, respectively. ${H2}_{n-t}$ and ${C2}_{n-t}$ denote the hidden state information and memorized state information from $\omega_{n-t-1}$ to $\omega_{n-t}$, respectively.

In order to extract all the information in the sentence related to the NER task and the RE task, we fuse ${H1}_{t+1}$, ${C1}_{t+1}$, ${H2}_{n-t}$, and ${C2}_{n-t}$, thus obtaining the fusion state information of the sentence $x_{t}$. The specific formula is shown in Equation (4).

(4) $$\begin{array}{c} x_{t}={H1}_{t+1}+{C1}_{t+1}+{H2}_{n-t}+{C2}_{n-t} \end{array}$$

#### 3.2.2. Separator

Since the fusion state information X = $x_{1}x_{2}\ldots x_{n}$ contains information related both to the NER task and the RE task, which in most cases will contain each other, it is difficult to extract these two types of information independently. Therefore, we adopt the idea of separation to separate the fusion state information into three types of features, namely features related only to NER, $\mu_{e}$, shared features, $\mu_{s}$, and features related only to RE, $\mu_{r}$. The exact formula is shown in Equation (5).

(5) $$\begin{array}{cc} \; & \mu_{e}=X\left[0,1/3\right]\\ \; & \mu_{s}=X\left[1/3,2/3\right]\\ \; & \mu_{r}=X\left[2/3,n\right] \end{array}$$

where X[0,1/3] denotes the features in the first one-third of the fused state information; $X\left[1/3,2/3\right]$ denotes the features in the middle one-third of the fused state information; and $X\left[2/3,n\right]$ denotes the features in the last one-third of the fused state information.

Since the features associated with the NER task contain both $\mu_{e}$ and $\mu_{s}$, and the features associated with the RE task include both $\mu_{r}$ and $\mu_{s}$, for each sub-task, we use a splicing operation. The two features are spliced and finally the features $H_{ner}$ related to the NER task and $H_{re}$ related to the RE task are obtained. The exact formula is shown in Equation (6).

(6) $$\begin{array}{cc} \; & H_{ner}=W_{es}\left(\mu_{e}:\mu_{s}\right)+b_{es}\\ \; & H_{re}=\;W_{sr}\left(\mu_{s}:\mu_{r}\right)+b_{sr} \end{array}$$

### 3.3. Named Entity Recognition Generation (NERG) Module

**Figure 4:**
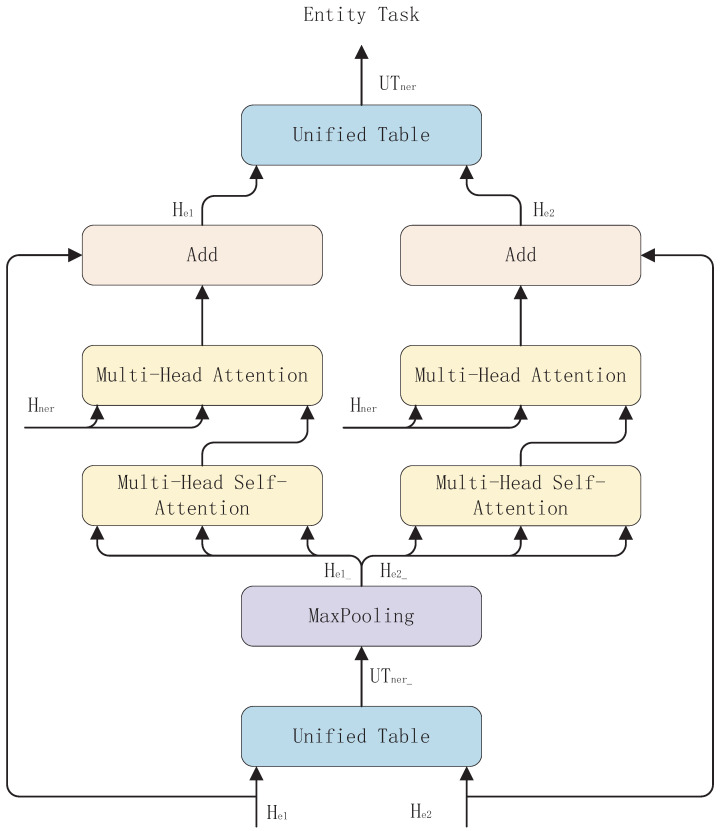
Named Entity Recognition Generation (NERG) module.

The NERG module is shown in Figure 4. In order to better extract all entities in a sentence, we use a feature $H_{e1}$ associated with the start boundary of the entity and a feature $H_{e2}$ associated with the end boundary of the entity to represent the boundary information of all entities in the sentence. In order to target the boundary information of entities more accurately, we adopt the design idea of the decoder in the Transformer [23] model to capture the maximum features of entity boundary information, $H_{e1\_}$ and $H_{e2\_}$, as well as to allow them to be associated with the features $H_{ner}$ that are relevant to the task of NER. The specific flow of the module is as follows.

First, in order to interact the feature $H_{e1}$ associated with the start boundary of the entity and the feature $H_{e2}$ associated with the end boundary of the entity, we apply the Hadamard product operation to $H_{e1}$ as well as $H_{e2}$ to generate a unified table feature of entity boundary information ${\mathrm{UF}}_{ner}$. The exact formula is shown in Equation (7).

(7) $$\begin{array}{c} {UF}_{ner}\left(i,j\right)=W_{ner}\left(H_{e1,i}•H_{e2,j}\right)+b_{ner} \end{array}$$

where • represents the Hadamard product, and $H_{e1,i}$ and $H_{e2,j}$ are the feature representations of the tokens $\omega_{i}$ and $\omega_{j}$, respectively.

Then, in order to capture the maximum association of the entity boundary information with the NER features, we use the maximum pooling operation to extract the maximum entity boundary features from the unified table features. The exact formula is shown in Equation (8).

(8) $$\begin{array}{cc} \; & H_{e1\_}=W_{e1\_}{maxpool}_{e1}\left({UF}_{ner}\right)+b_{e1\_}\\ \; & H_{e2\_}=W_{e2\_}{maxpool}_{e2}\left({UF}_{ner}\right)+b_{e2\_} \end{array}$$

Next, we adopted a design idea based on the decoder in Transformer [23]. Multi-head self-attention is first used to capture the maximum intrinsic association of entity boundaries between entities in a sentence. Then, we use the multi-head attention method to allow the maximum entity boundary information in the sentence to fully interact with the features $H_{ner}$ that are relevant to the task of NER to mine the information that can target the entity boundary in the NER task. Finally, we fuse the obtained information with the original entity boundary information to obtain the new entity boundary information. The specific formula is shown in Equation (9).

(9) $$\begin{array}{cc} \; & H_{e1\_/e2\_}=MultiHeadSelfAtt\left({UF}_{ner}\right)\\ \; & H_{e1\_/e2\_}=MultiHeadAtt\left(H_{e1\_/e2\_},H_{ner},H_{ner}\right)\\ \; & H_{e1/e2}=H_{e1/e2}+H_{e1\_/e2\_} \end{array}$$

Finally, we again use the Hadamard product operation to obtain the unified table features ${\mathrm{UF}}_{ner\_}$ of the entity boundary information and perform table filling to generate the NER task. The specific formula is shown in Equation (10).

(10) $$\begin{array}{cc} \; & {UF}_{ner\_}\left(i,j\right)=W_{ner\_}\left(H_{e1,i}•H_{e2,j}\right)+b_{ner\_}\\ \; & \hat{{table}_{ner}}\left(i,j\right)=softmax\left(ReLU\left({UF}_{ner\_}\right)\right)\\ \; & {table}_{ner}\left(i,j\right)={argmax}_{l\in L_{ner}}\left(\hat{{table}_{ner}}\left(i,j\right)\right) \end{array}$$

where $\hat{{table}_{ner}}\left(i,j\right)$ denotes the initial table features for the named body recognition task, and ${table}_{ner}\left(i,j\right)$ denotes the labeling results of the entities $\omega_{i\cdots j}$.

### 3.4. Relation Extraction Generation (REG) Module

**Figure 5:**
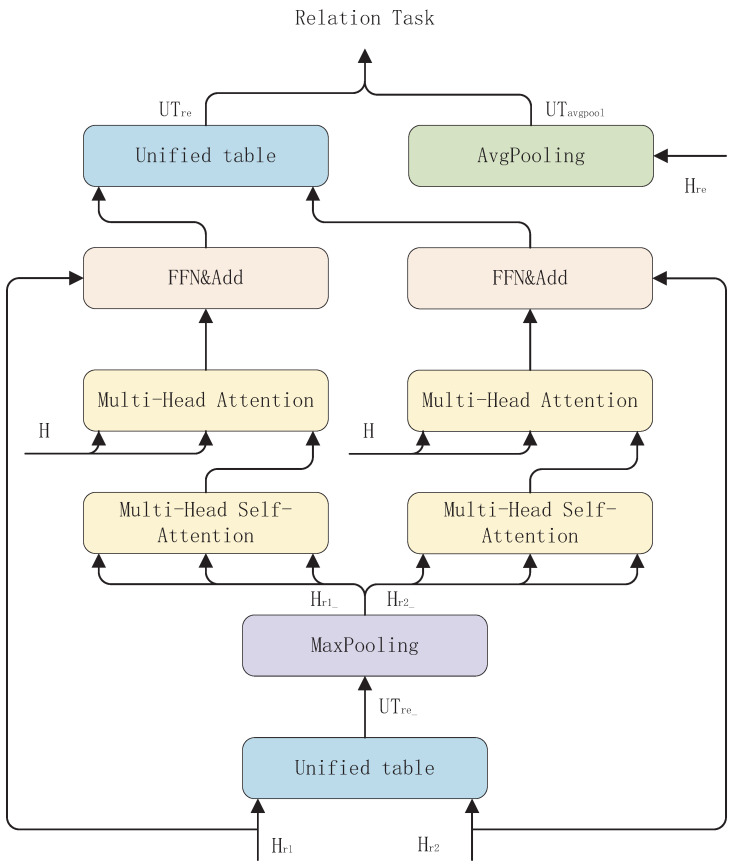
Relation Extraction Generation (REG) module.

The REG module is shown in Figure 5. We use the features $H_{r1}$ and $H_{r2}$ to represent the entity pair start boundary and entity pair end boundary for each relation in the sentence. In order to be able to target the entity pair boundary information corresponding to each relation more accurately, we adopt the design idea of the decoder in the Transformer [23] model to capture the association of the entity pair boundary information maximal features $H_{r1\_}$ and $H_{r2\_}$ of each relation with the sequence of sentence token representations H. In addition, in order to fuse the features associated with the RE task $H_{re}$, we use an average pooling operation to fuse $H_{re}$ into each table entry in the RE task. The specific flow of the module is as follows.

First, in order to correlate the entity-pair start boundary information and entity-pair end boundary information with each other, we perform a Hadamard product operation on $H_{r1}$ and $H_{r2}$ to generate a table of entity-pair boundary information (i.e., a unified table feature) ${UF}_{re}$ for each relation in the sentence. The specific formula is shown in Equation (11).

(11) $$\begin{array}{cc} \; & {UF}_{re}\left(i,j,r\right)=W_{re}\left(H_{r1,i,r}•H_{r2,j,r}\right)+b_{re} \end{array}$$

where • represents the Hadamard product, and $H_{r1,i,r}$ and $H_{r2,j,r}$ denote the feature representations of tokens $\omega_{i}$ and $\omega_{j}$ of relation r, respectively.

Then, since the determination of the boundary information of subject and object in the relational triple is closely related to the semantic information of the sentence, we adopt the maximum pooling operation here to extract the maximum boundary information from the entity hidden in ${UF}_{re}$. The specific formula is shown in Equation (12).

(12) $$\begin{array}{cc} \; & H_{r1\_}=W_{r1\_}{maxpool}_{r1}\left({UF}_{re}\right)+b_{r1\_}\\ \; & H_{r2\_}=W_{r2\_}{maxpool}_{r2}\left({UF}_{re}\right)+b_{r2\_} \end{array}$$

Next, we use the same idea based on the decoder in Transformer [23]. Multi-head self-attention is first used to capture the interconnection of entity pair boundary information between relational triples in a sentence. Then, we use a multi-head attention method to allow the maximum entity pair boundary information in the sentence to interact sufficiently with the sequence of sentence token representations H to more accurately target the entity pair boundaries of each relational triple in the sentence. Finally, we fuse the obtained information with the original entity pair boundary information to become the new entity pair boundary information. The specific formula is shown in Equation (13).

(13) $$\begin{array}{cc} \; & H_{r1\_/r2\_}=MultiHeadSelfAtt\left({UF}_{re}\right)\\ \; & H_{r1\_/r2\_}=MultiHeadAtt\left(H_{r1\_/r2\_},H,H\right)\\ \; & H_{r1/r2}=H_{r1/r2}+H_{r1\_/r2\_} \end{array}$$

Finally, since the new entity pair boundary information does not fuse the feature $H_{re}$, which is relevant to the RE task, we apply an average pooling operation to $H_{re}$ to compress its embedded feature information into a single word. Finally, it is fused into each table entry in the new unified table feature ${\mathrm{UF}}_{re\_}$, and table filling is performed for each table entry to generate the RE task. The specific formula is shown in Equation (14).

(14) $$\begin{array}{cc} \; & H_{avg}=W_{avg}{avgpool}_{re}\left(H_{re}\right)+b_{avg}\\ \; & {UF}_{avg\_}\left(i,j,r\right)=W_{avg\_}\left(H_{avg\_,i,r}•H_{avg\_,j,r}\right)+b_{avg\_}\\ \; & {UF}_{re\_}\left(i,j,r\right)=W_{re\_}\left(H_{r1\_,i,r}•H_{r2\_,j,r}\right)+b_{re\_}\\ \; & \hat{{table}_{re}}\left(i,j,r\right)=softmax\left(ReLU\left({UF}_{re\_}+{UF}_{avg\_}\right)\right)\\ \; & {table}_{re}\left(i,j,r\right)={argmax}_{l\in L_{re}}\left(\hat{{table}_{re}}\left(i,j,r\right)\right) \end{array}$$

where $\hat{{table}_{re}}\left(i,j,r\right)$ denotes the initial table features for the relation extraction task, and ${table}_{re}\left(i,j,r\right)$ denotes the labeling results of token pairs ($\omega_{i}$,$\omega_{j}$) for relation r.

### 3.5. Loss Function

The loss function of our model is as follows. For each given training set, the loss function L that guides the model during training consists of two parts: $L_{ner}$ denotes the loss function for the NER task and $L_{re}$ denotes the loss function for the RE task. In addition, we perform a Sigmoid operation on the values of $L_{ner}$ and $L_{re}$ to dynamically control the learning weights of the NER task and the RE task.

(15) $$\begin{array}{cc} \; & L_{ner}=-\sum_{i=1}^{n}\sum_{j=1}^{n}y_{i,j}logP_{i,j}\\ \; & L_{re}=-\sum_{i=1}^{n}\sum_{j=1}^{n}\sum_{r=1}^{\left|R\right|}y_{i,j,r}logP_{i,j,r}\\ \; & L={\frac{exp(L_{ner})}{exp\left(L_{ner}\right)+exp\left(L_{re}\right)}L}_{ner}+{\frac{exp(L_{re})}{exp\left(L_{ner}\right)+exp\left(L_{re}\right)}L}_{re} \end{array}$$

where (*i*, *j*) denotes the index of ($\omega_{i}$, $\omega_{j}$) labels in the NER task; (*i*, *j*, *r*) denotes the index of ($\omega_{i}$,$\omega_{j}$) labels with relation *r* in the RE task; and both $L_{ner}$ and $L_{re}$ use the cross-entropy loss function.

## 4. Experiments

### 4.1. Experimental Settings

#### 4.1.1. Datasets

We evaluated our model on the ACE2005 [24] dataset as well as the SciERC [25] dataset. The ACE2005 dataset was collected from a variety of sources, including news articles and online forums. This dataset was built on top of the ACE2004 dataset and is often used as a benchmark test for NER and RE methods. In the ACE2005 dataset, seven entity categories were defined and six relation categories were defined for each pair of entities. The SciERC dataset is derived from 500 abstracts taken from papers in the field of artificial intelligence, which include annotations on scientific entities, their relations, and co-reference clusters. The dataset is predefined with six scientific entity types and seven relation types. The purpose of this dataset is to provide a benchmark test dataset for evaluating the performance of NER and RE tasks. The specific content distribution of these two datasets is shown in Table 1.

#### 4.1.2. Evaluation Metrics

We use precision, recall, and micro-F1 as our evaluation metrics. For NER, a prediction is considered correct only if the predicted entity boundaries as well as types match the ground truth exactly; for RE, a prediction is considered correct only if the predicted entity boundaries as well as relation types in the relational triple match the ground truth exactly; and for RE+, a prediction is considered correct only if the predicted entity boundaries and entity types as well as relation types in the relational triple match the ground truth exactly. In addition, for a fair model comparison, we discuss only the case where the encoder is BERT-Base-Cased [22] on the ACE2005 dataset, and only the case where the encoder is SciBERT [26] on the SciERC dataset.

#### 4.1.3. Baselines

We compare the model with the following joint entity and relation extraction models: SPE [27], MRC4ERE++ [28], TRIMF [18], UNIRE [29], PURE [30], PFN [8], TablERT [9], TablERT-CNN [10], MGE [19], and PL-Marker [31].

Most of the experimental results of these baseline models were copied directly from their original papers.

#### 4.1.4. Implementation Details

Our experiments were carried out on an Ubuntu 18.04.6 LTS workstation with a single A40. We used the Adam [32] optimizer for model training. The learning rate was 1 × $10^{-5}$ on the ACE2005 dataset and 3 × $10^{-5}$ on the SciERC dataset. The number of training epoch was 100. The batch size of the training set was set to 4. The batch size of the validation and test sets was set to 6. We set the maximum length of the input sentence to 100. The other parameters were randomly initialized.

### 4.2. Main Experimental Results

Table 2 demonstrates the performance comparison of our model with other benchmark models. From Table 2, it can be seen that our model NER’s F1 scores on the ACE2005 dataset and SciERC dataset are 0.4% and 0.4% lower than the F1 scores of the best model, respectively. However, our model achieved optimality on RE and RE+. This is due to the fact that previous models have focused more on the performance enhancement of the NER task and have not fully explored the effect of the subtask interaction balance between the entity and relation on the relational triple extraction. We set up the FSN module to separate the features related only to the NER task and the features related only to the RE task, so as to accomplish the NER task and the RE task so that they fully interact with each other, and then to achieve the intrinsic correlation between entity and relation. This is a testament to the strength of our FSN module design.

Compared to the joint entity and relation extraction model PFN, which is also based on table filling, our model achieved absolute performance gains on both the ACE2005 dataset and the SciERC dataset. We attribute this performance improvement to the NERG and REG modules we set up. The NERG and REG modules can more accurately target all entity boundaries contained in a sentence and entity pair boundaries in a relational triple, respectively. In addition to this, we explored the performance differences between the pipeline-based method and the joint entity and relation extraction method. Compared to the PURE model using the pipeline-based method, our model achieved performance improvements of 2.9%, 6.0%, and 6.7% for the F1 scores of NER, F1 scores of RE, and F1 scores of RE+ on the SciERC dataset, respectively. In addition to the reason that joint entity and relation extraction can solve the subtask independence problem as well as the error propagation problem, we attribute this performance improvement to the setup of the FSN module. The FSN module interacts with the NER task and the RE task by setting up a shared partition, which solves the problems of difficult interaction between subtasks and error propagation in conventional pipeline-based methods.

### 4.3. Ablation Study

In this section, we explore the impact of each part of our model on RE+. Some of the parts of the model explored include the FSN module (Forward_LSTM, Backward_LSTM), NERG module (NER_MFE), and REG module (RE_MFE, RE_AvgPooling).

We mainly explore the effect of forward LSTM filtered sentence information and reverse LSTM filtered sentence information on balancing subtask interactions in the FSN module. As shown in Table 3, removing the forward LSTM and backward LSTM in the FSN module reduces the RE+ scores by 3.5% and 3.8%, respectively. This is because the hidden state information in LSTM captures the information of the current time step and passes this information to the next time step, and enables continuous modeling of sequence data, whereas the memory state information controls the flow and retention of information, which allows the model to selectively forget and retain the information, thus enabling the capture of long-term dependencies as well as better prediction of future sequences. Removing forward LSTM and backward LSTM will result in that word-to-word hidden state information and word-to-word memory state information will not be captured.

When we removed the NERG module, we found that RE+ scores dropped by 3.6%. This huge performance gain is attributed to the NERG module’s ability to capture the maximum correlation between sentence semantic information and entity pair boundary information by fully utilizing Hadamard product operations and attention mechanisms, which enables better extraction of entity pair boundary information in relational triples.

Similarly, we explored the impact of removing the local feature extraction part of the REG module on RE task performance. As can be seen in Table 3, the performance has decreased by 4.1%. This indicates that the local feature extraction part of the REG module can contribute to capture the entity pair boundary information corresponding to each relation in a sentence. In addition to this, we performed an ablation study on the maximum pooling operation in the REG module. In Table 3, it is shown that removing the maximum pooling operation in the REG module decreases the RE+ performance by 3.3%. The main reason for this is that the features related to the RE task contain associations between entity and relation in a relational triple. This association is incorporated into each table entry of the RE task through the average pooling operation, which improves the performance of RE+.

### 4.4. Robustness Test on Named Entity Recognition

We use robustness tests to evaluate the stability of our model in the face of various special cases. The performance of our model as well as the baseline model in the face of a NER-facing dataset domain transformation method proposed by Wang et al. [33] is demonstrated in Table 4, and the specific transformation method is shown at https://www.textflint.io/. We compare our model with several unrelated models, including the BiLSTM-CRF model [34], the BERT model [22], the TENER model [35], and the Flair Embeddings model [36].

Based on the observations in Table 4, we can find that our model exhibits greater robustness in the face of input perturbations compared to other baseline models, especially in the case of cross-categories. This increase in robustness may be attributed to the fact that we use relational signaling of type-constrained entities in our training. In our model, reasoning about entity types is not only influenced by the semantic meaning of the target entity itself, but also by the (relation) context around the entity. This means that our model takes into account the contextual information around the entity when reasoning about entity types, rather than relying only on the characteristics of the entity itself. This type-constrained training approach allows our model to better understand the relationship between an entity and its surroundings, which improves its robustness in the presence of input perturbations. When confronted with cross-category situations, where the type of an entity does not exactly match the type of other entities in its surroundings, our model is better able to adapt to and deal with this complexity.

### 4.5. Model Efficiency

We evaluate the training time as well as the inference time for the efficiency of our model mainly with PFN, a joint entity and relation extraction model that also employs a table-filling method. The results in Table 5 demonstrate that while both our model and the PFN model are theoretically O(N$L^{2}$), our model took less time to train on the ACE2005 dataset as well as the SciERC dataset. We attribute this improvement in model training efficiency to the FSN module in our model. Compared to the previous joint entity and relation extraction models, the FSN module makes it simpler to perform feature extraction for all subtasks, as well as making it simpler to accomplish subtask interactions through partitioning operations by extracting the hidden state information and memory state information from each word to the next in a sentence. Although the model required similar model inference time on both datasets, our model achieved 7.1% and 3.9% performance improvement over the PFN model on the ACE2005 dataset as well as the SciERC dataset, respectively, which is sufficient to demonstrate the advantages of our model design.

## 5. Conclusions

In this paper, we mainly analyze the advantages and disadvantages of joint learning methods based on shared parameters and joint learning methods based on joint decoding and propose a new joint entity and relation extraction method, which sets up a FSN module to solve the problem of interaction imbalance among subtasks by adopting the filter and separator as well as splicing operation. We also set up a NERG module and a REG module to solve the problem of insufficient extraction of local features from subtasks by adopting the design idea of the decoder in Transformer and a pooling operation. In addition, we propose a dynamic loss function for model optimization. We conducted comprehensive experiments on two public datasets, demonstrating that our model yields more desirable outcomes compared to the baseline model. Further analyses and ablation studies validate the significance of every modular component in our model.

## Figures and Tables

**Table 1:** Statistics of datasets. |E| and |R| denote the number of entity types and the number of relation types, respectively.

Dataset	Sentences			Entities	Relations	|E|	|R|
	**Train**	**Dev**	**Test**				
ACE2005	10,051	2424	2050	38,287	7070	7	6
SciERC	1861	275	551	8094	4684	6	7

**Table 2:** Results of the main experiments on the ACE2005 and SciERC datasets. * denotes results generated from source code. ^♢^ denotes that the model leverages the cross-sentence information. The best results are shown in bold and the second-best results are underlined. BERT-Based-Cased [22] and SciBERT [26] were used on the ACE2005 and SciERC datasets, respectively.

Dataset	Model	NER	RE	RE+
ACE2005	SPE	87.2	66.7	63.2
	MRC4ERE++	85.5	-	62.1
	${TriMF}^{\;♢}$	87.6	66.5	62.8
	PFN	85.5 *	-	58.6 *
	PURE	**88.7**	66.7	63.9
	TablERT	87.6	66.2	62.6
	TablERT-CNN	87.8	65.0	61.8
	FSN	88.3	**68.7**	**65.7**
SciERC	SPE	68.0	47.6	34.6
	${UniRE}^{\;♢}$	68.4	-	36.9
	PURE	66.6	48.2	35.6
	PFN	66.8	-	38.4
	MGE	68.4	-	39.4
	${PL-Marker}^{\;♢}$	**69.9**	53.2	41.6
	FSN	69.5	**54.2**	**42.3**

**Table 3:** Ablation study of FSN on ACE2005. The best of these experimental results are marked in bold.

Albation	Pre.	Rec.	F1
FSN	**70.0**	**61.9**	**65.7**
w/o Forward_LSTM	67.8	57.5	62.2
w/o Backward_LSTM	66.0	58.2	61.9
w/o NER_MFE	66.4	58.3	62.1
w/o RE_MFE	65.3	58.2	61.6
w/o RE_AvgPooling	66.7	58.7	62.4

**Table 4:** Robustness test of NER against input perturbation in ACE2005; baseline results and test files are copied from https://www.textflint.io/ (accessed on 30 December 2023).

Model	ConcatSent		CrossCategory		EntTypos		OOV		SwapLonger		Average
	Ori→Aug	Decline	Ori→Aug	Decline	Ori→Aug	Decline	Ori→Aug	Decline	Ori→Aug	Decline	Decline
BiLSTM-CRF	83.0→82.2	0.8	82.9→43.5	39.4	82.5→73.5	9.0	82.9→64.2	18.7	82.9→67.7	15.2	16.6
BERT-based (cased)	87.3→86.2	1.1	87.4→48.1	39.3	87.5→83.1	4.1	87.4→79.0	8.4	87.4→82.1	5.3	11.6
BERT-based (uncased)	88.8→88.7	**0.1**	88.7→46.0	42.7	89.1→83.0	6.1	88.7→74.6	14.1	88.7→78.5	10.2	14.6
TENER	84.2→83.4	0.8	84.7→39.6	45.1	84.5→76.6	7.9	84.7→51.5	33.2	84.7→31.1	53.6	28.1
Flair	85.5→85.2	0.3	84.6→44.9	39.7	86.1→81.5	4.6	84.6→81.3	**3.3**	84.6→73.1	11.5	11.9
PFN	89.1→87.9	1.2	89.0→80.5	8.5	89.6→86.9	**2.7**	89.0→80.4	8.6	89.0→84.3	4.7	5.1
FSN	88.3→86.4	1.9	88.3→82.7	**5.6**	88.8→86.2	**2.7**	88.3→81.1	7.2	88.3→85.6	**2.7**	**4.0**

**Table 5:** Comparison of the model efficiency. Training time (s) refers to the amount of time needed to train one epoch.; inference time (ms) is the amount of time it takes to predict relational triples of a single sentence. * denotes results acquired from the source code.

Dataset	Model	Training Time	Inference Time	F1
ACE2005	PFN	361	17	58.6 *
	FSN	**319**	**16**	**65.7**
SciERC	PFN	74	6	38.4
	SOIRP	**65**	**3**	**42.3**

## Data Availability

The data presented in this study are available upon request from the corresponding author. The data are not publicly available due to copyright.
